# Supplementary figures and images for: Genomic bases underlying the adaptive radiation of core landbirds
Source: BMC Ecol Evol. 2021 Aug 28;21:162. doi: 10.1186/s12862-021-01888-5 (PMC8403425; doi:10.1186/s12862-021-01888-5)

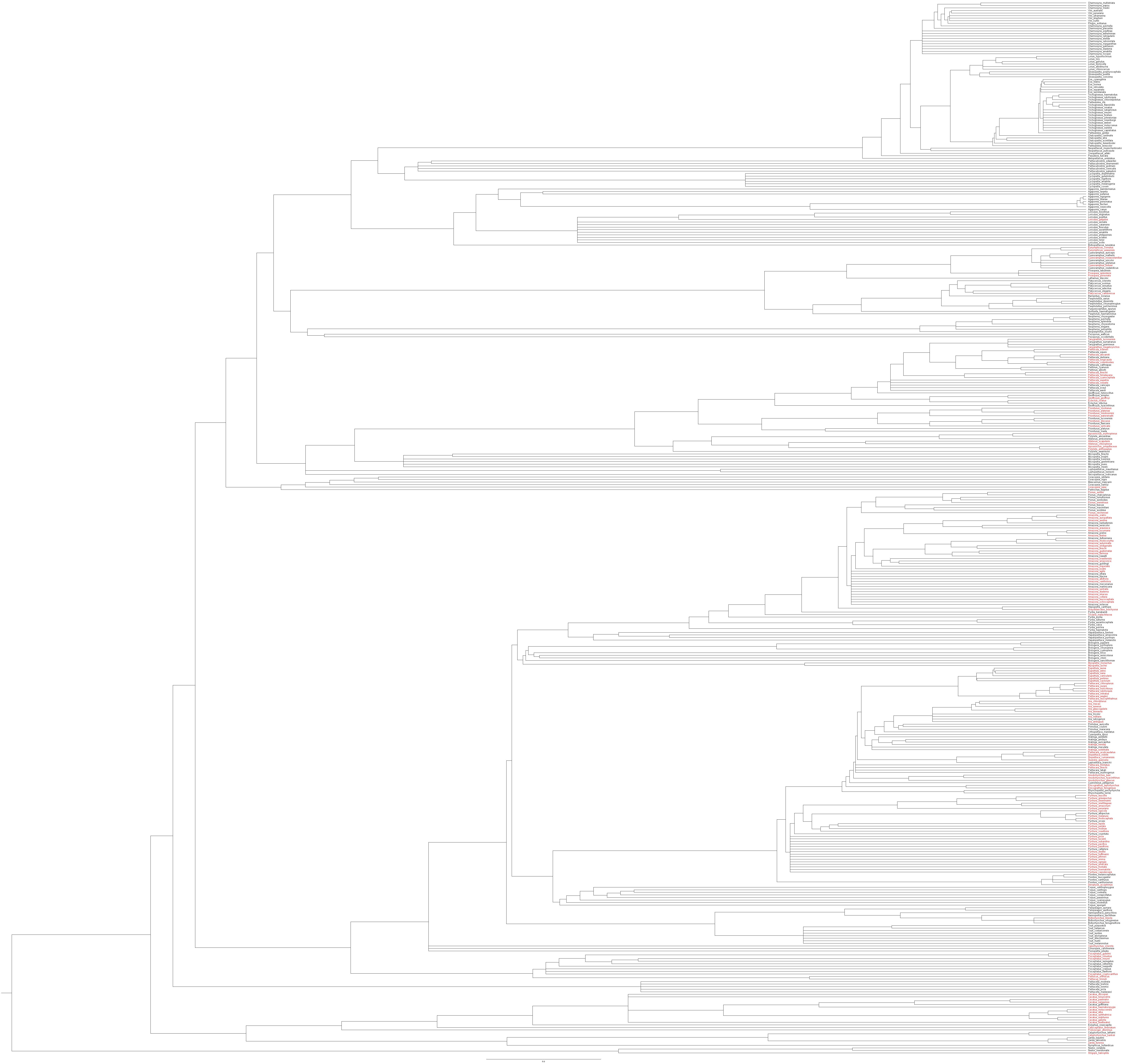

Supplement: Supplementary file 12 — Additional file 12: Fig. S1 Phylogenetic supertree of 413 parrot species. Parrots that consume nuts in their diets are shown in red. The phylogenetic supertree and dietary information follow one published study (Burgio et al. [47]) [file 12862_2021_1888_MOESM12_ESM.jpg]
